# Impact of acute pancreatitis on the outcomes of cholecystectomy. An observational multicenter retrospective study

**DOI:** 10.1007/s00423-025-03859-6

**Published:** 2025-10-03

**Authors:** Łukasz Nawacki, Magdalena Kołomańska, Robert Mazurkiewicz, Marcin Niżnik, Krzysztof Ratnicki, Małgorzata Węsierska, Piotr Myrcha, Jerzy Zabłocki, Kryspin Mitura, Laura Kacprzak, Małgorzata Pajer, Piotr Richter, Kamil Rapacz, Maciej Sroczyński, Mateusz Szmit, Iwona Gorczyca-Głowacka

**Affiliations:** 1 Collegium Medicum, The Jan Kochanowski University, Kielce, 25-317 Poland; 2 Department of General Surgery, Independent Public Health Care Institution of the Ministry of Internal Affairs and Administration, Gdańsk, Poland; 3 Department of General and Vascular Surgery, Faculty of Medicine, Medical University of Warsaw, Warsaw, 02-091 Poland; 4 University in Siedlce, Siedlce, 08-110 Poland; 5 Siedlce Hospital, Siedle, 08-110 Poland; 6 First Department of General Surgery, Jagiellonian University Medical College, Cracow, 30-688 Poland; 7 Doctoral School of Medical and Health Sciences, Jagiellonian University Medical College, Cracow, 30-688 Poland; 8 Department of General, Minimally Invasive and Endocrine Surgery, Wroclaw Medical University, Wroclaw, 50-556 Poland

**Keywords:** Acute pancreatitis, Laparoscopic cholecystectomy, Complications, Pancreatitis, Cholecystectomy

## Abstract

**Purpose:**

Gallstones are the most common cause of acute pancreatitis (AP), which usually necessitate hospitalization. Although cholecystectomy is required to prevent recurrent episodes, no clear guidelines have been established yet regarding the proper timing of cholecystectomy after an AP incidence. The objective was to evaluate the impact of AP on the course of cholecystectomy procedures, both by comparing patients with and without a history of AP and by analyzing the AP subgroup according to timing and severity.

**Methods:**

This retrospective evaluation was performed on patients who had undergone cholecystectomy for various causes in six hospitals in Poland. Patients were divided into the following three groups: patients without AP, patients with a history of AP, and patients with active AP. The analyzed variables included the surgical type and duration, postoperative complications, AP severity, and time elapsed between cholecystectomy and AP treatment completion.

**Results:**

In total, 4183 patients were included in the study, including 3948 without AP, 209 with a history of AP, and 26 with active AP. The most common surgical indications in patients with active AP and history of AP were concomitant cholecystitis (53.85%) and cholelithiasis (82.3%), respectively. The mean complication rates were not significantly different between groups. The surgical type was significantly affected by AP severity. Surgery in the period between 15 and 31 days post-AP attack was associated the highest frequency of minimally invasive surgery and shortest surgical duration, although the differences were not significant.

**Conclusion:**

Inflammation is the most common associated cause of surgery during the course of active AP. Surgery at 15–31 days post-AP attack was associated with a higher frequency of minimally invasive surgery and shorter operative duration, although these differences did not reach statistical significance. Our findings therefore suggest, but do not confirm, that this may represent a favorable time window, which warrants further evaluation in prospective studies.

## Introduction

In 2014, a total of 77,354 cholecystectomies were performed in Poland; by 2017, this number increased to 80,159 [1]. The incidence rates are expected to continue to increase worldwide due, among other things, to the global epidemic of obesity, which represents a major risk factor for the development of cholelithiasis. The other risk factors include excessive caloric and animal fat intake, hyperlipidemia, oral hormonal contraception, prolonged food deprivation, as well as rapid weight loss and infectious factors [2]. One of the severe complications of gallstones is acute pancreatitis (AP) [3], a disease which frequency is also increasing steadily. The gold standard for the treatment of symptomatic gallbladder stones is cholecystectomy, either open surgical or laparoscopic, with laparoscopic cholecystectomy being the preferred approach [4]. However, to date, no unified international guidelines are available regarding the indications of cholecystectomy in case of gallstones. Although patients with symptomatic gallstones should be subjected to surgical treatment, up to 22% of patients experience abdominal pain postoperatively [5]. In a previous study assessing the usefulness of a watchful waiting strategy versus surgical management in patients with gallstones [6], only the patients with typical biliary pain symptoms were more likely to have no pain after surgery, as compared to patients with other symptoms.

The various factors associated with an increased risk of AP in patients with gallstones are as follows: gallstone size of < 5 mm, gallbladder duct width of > 5 mm, and the number of stones of > 20 [7]. Given that AP could be the initial presentation of gallstones, there is no consensus regarding when to remove the gallbladder and whether elective cholecystectomy should be performed in asymptomatic patients presenting with the aforementioned risk factors for AP. Likewise, no consensus has been reached regarding the management of AP occurring as a complication of biliary stones. The initial trigger for AP occurrence in patients with biliary stones is the blockage of bile and pancreatic juice outflow by impacted bile stones or sludge. Therefore, it would seem logical to perform an incision (sphincterotomy) to unblock the ampulla of Vater to relieve pressure in the pancreatic and bile ducts. However, performing endoscopic retrograde cholangiopancreatography (ERCP) in the early stages of acute pancreatitis is still controversial. A large meta-analysis [8] failed to prove that early ERCP considerably improves the rates of mortality or morbidity, including local and systemic complications, in patients with AP. Therefore, currently in AP cases with accompanying biliary stones, ERCP is recommended in patients with concomitant cholangitis or biliary dilatation [9]. Contrarily, ERCP alone does not address the cause of AP, as it only removes the impacted choledochal stones while the gallbladder with its stones is still remaining; hence, there is a potential risk for recurrent attacks of AP. Some experts [10, 11] believe that early cholecystectomy in mild AP cases has advantages over distant cholecystectomy. Similarly, the Japanese Guidelines for the management of acute pancreatitis emphasize early diagnosis and appropriate timing of intervention, underscoring the ongoing international debate regarding optimal surgical timing [12]. However, early cholecystectomy may increase the risk of complications [13].

This multicenter retrospective study provides a cross-sectional overview of gallbladder surgery in Poland. The primary analysis focused on patients with acute pancreatitis, stratified by timing and severity, to evaluate outcomes of early versus delayed cholecystectomy in line with current clinical guideline considerations. To provide context, patients without AP were also included as a baseline comparator group, enabling us to quantify how AP (active or past) influences surgical technique, timing, and complication risk relative to standard cholecystectomy. Accordingly, the study had two main aims: [1] to compare outcomes of cholecystectomy in patients with and without a history of AP, and [2] to perform subgroup analyses among AP patients stratified by severity and timing of surgery.

## Materials and methods

This multicenter observational retrospective study was conducted at six surgical centers (four teaching hospitals and two provincial hospitals) in Poland from 2019 to 2022. The study was conducted in accordance with the Helsinki standards and was adherent to the STROBE criteria for retrospective studies. The requirement for obtaining the participants’ consent was waived owing to the retrospective design of the study. All data has been fully anonymized. Approval for the study had been obtained from the Bioethics Committee of Jan Kochanowski University in Kielce (decision no. 54/2022 dated 04.11.2022). Data was accessed and collected between 01.01.2023 and 31.03.2023.

The study group comprised patients who underwent cholecystectomy procedures during the target period. The inclusion criteria were as follows: age of > 18 years and undergoing cholecystectomy, regardless of the surgical indication (e.g., biliary colic, cholecystitis, gallstone pancreatitis, or other causes) or surgical type. This broad approach was chosen to reflect real-world surgical practice and to capture the entire spectrum of patients who undergo cholecystectomy, thereby allowing us to compare outcomes in those with and without acute pancreatitis in a clinically representative cohort. Surgical indications were subsequently stratified in the analysis to account for this heterogeneity.

The collected data included the patients’ age, surgical indication, mode of surgery (laparoscopy, laparotomy, or laparoscopy with conversion), episodes of AP (current or past), AP severity based on the Atlanta 2012 classification [14], cause of AP, time between surgery and the last AP episode, intraoperative and postoperative complications, surgical duration, postoperative abdominal drainage, and histopathological examination findings. According to the Atlanta 2012 classification, patients were divided into three groups of severity [14]:

- Mild – no organ failure and no local or systemic complications.
- Moderately severe – transient organ failure, local complications, or exacerbation of comorbid disease.
- Severe – persistent organ failure (> 48 h).

AP severity was retrospectively classified according to the Atlanta 2012 criteria, based on information available in patient records. Organ failure was identified when documentation reported respiratory, cardiovascular, or renal dysfunction, but the duration (> 48 h) could not always be verified with certainty. Likewise, information on local complications (e.g., necrosis, fluid collections) was not consistently available, and patients were categorized according to the best available clinical data. Patients for whom no information on disease course was available were excluded from further analysis.

### Statistical analysis

Qualitative (i.e., non-numerical) variables were expressed as frequencies and percentages and were compared between groups by chi-squared test (with Yates continuity correction for 2 × 2 tables) or the Fisher’s exact test for low expected counts within the tables. Quantitative variables (i.e., numerical) were expressed as mean and standard deviation (SD) and were compared between two groups using the Mann–Whitney’s test. The comparison of quantitative variables among three or more groups was carried out using the Kruskal–Wallis test. If significant differences between groups were detected, Dunn’s post-hoc test was performed to determine the significantly different groups. The significance level was established at 0.05; consequently, all *p*-values < 0.05 were interpreted as indicative of significant relationships. Statistical analysis was performed by using R software, version 4.3.1 [15].

### Results

Altogether, 4,183 patients were included in the study, with 70% of them being female. The patients’ mean age was 55.1 years, and the mean BMI was 28.6 kg/m^2^. Only 25.6% of the patients had normal BMI and 38.3% were overweight. The remaining patients were obese, with 24.8%, 7.9%, and 3.4% of the patients having obesity classes 1, 2, and 3, respectively. Cholecystolithiasis was an indication for surgery in 3,177 (76%) patients.

Among the analyzed patients, 26 were operated on during the course of active AP, 209 had a history of AP, and the remaining 3,948 had no AP. The characteristics of the included patients are summarized in Table 1.

**Table 1:** The characteristics of the patients stratified according to their AP status

Parameter		No AP (*N* = 3948) − A	Active AP (*N* = 26) − B	History of AP (*N* = 209) − C	Total (*N* = 4183)	*p*-value
Indications for surgery **	Cholelithiasis	2993 (75.81%)	12 (46.15%)	172 (82.30%)	3177 (75.95%)	*p* < 0.001 *
	Inflammation	874 (22.14%)	14 (53.85%)	42 (20.10%)	930 (22.23%)	*p* < 0.001 *
	Polyp	136 (3.44%)	0 (0.00%)	0 (0.00%)	136 (3.25%)	*p* = 0.005 *
	Tumor	34 (0.86%)	0 (0.00%)	0 (0.00%)	34 (0.81%)	*p* = 0.527
	Other	8 (0.20%)	0 (0.00%)	0 (0.00%)	8 (0.19%)	*p* = 1
Surgery mode	Elective	3228 (81.76%)	10 (38.46%)	187 (89.47%)	3425 (81.88%)	*p* < 0.001 *
	Emergency	720 (18.24%)	16 (61.54%)	22 (10.53%)	758 (18.12%)	
Surgery type	Laparoscopy	3564 (90.27%)	18 (69.23%)	183 (87.56%)	3765 (90.01%)	*p* = 0.002 *
	Laparotomy	261 (6.61%)	8 (30.77%)	17 (8.13%)	286 (6.84%)	
	Laparoscopy + conversion	123 (3.12%)	0 (0.00%)	9 (4.31%)	132 (3.16%)	
Duration of surgery [min]	Mean (SD)	68.01 (30.84)	70.38 (14.73)	74.08 (33.53)	68.29 (30.96)	*p* = 0.038 *
	Median (quartiles)	60 (45–80)	67.5 (59.5–76.25)	65 (50–85)	60 (47–80)	
	Range	15–455	55–100	25–234	15–455	C > A
Abdominal drainage	No	510 (12.92%)	0 (0.00%)	18 (8.61%)	528 (12.62%)	*p* = 0.02 *
	Yes	3430 (86.88%)	26 (100.00%)	191 (91.39%)	3647 (87.19%)	
	No data	8 (0.20%)	0 (0.00%)	0 (0.00%)	8 (0.19%)	
Histopathology result **	Gallbladder unremarkable	39 (0.99%)	0 (0.00%)	3 (1.44%)	42 (1.00%)	*p* = 0.591
	Chronic inflammation	3300 (83.59%)	19 (73.08%)	177 (84.69%)	3496 (83.58%)	*p* = 0.31
	Acute inflammation	327 (8.28%)	5 (19.23%)	20 (9.57%)	352 (8.42%)	*p* = 0.099
	Gangrenous inflammation	102 (2.58%)	2 (7.69%)	3 (1.44%)	107 (2.56%)	*p* = 0.141
	Cancer	30 (0.76%)	0 (0.00%)	0 (0.00%)	30 (0.72%)	*p* = 0.504
Postoperative complications: surgical **	None	3791 (96.02%)	24 (92.31%)	198 (94.74%)	4013 (95.94%)	*p* = 0.264
	Infection of the surgical site	51 (1.29%)	0 (0.00%)	1 (0.48%)	52 (1.24%)	*p* = 0.653
	Bile leak	29 (0.73%)	1 (3.85%)	2 (0.96%)	32 (0.77%)	*p* = 0.143
	Postoperative hemorrhage	32 (0.81%)	1 (3.85%)	3 (1.44%)	36 (0.86%)	*p* = 0.093
	Other	33 (0.84%)	0 (0.00%)	3 (1.44%)	36 (0.86%)	*p* = 0.541
	Reoperation	13 (0.33%)	0 (0.00%)	2 (0.96%)	15 (0.36%)	*p* = 0.246
Postoperative complications: cardiological **	None	3897 (98.71%)	25 (96.15%)	207 (99.04%)	4129 (98.71%)	*p* = 0.425
	Heart rhythm disturbances	28 (0.71%)	0 (0.00%)	1 (0.48%)	29 (0.69%)	*p* = 1
	Cardiac failure	15 (0.38%)	0 (0.00%)	1 (0.48%)	16 (0.38%)	*p* = 0.604
	Infarction	8 (0.20%)	1 (3.85%)	0 (0.00%)	9 (0.22%)	*p* = 0.063
Postoperative complications: pulmonary **	None	3913 (99.11%)	26 (100.00%)	208 (99.52%)	4147 (99.14%)	*p* = 1
	Pneumonia	19 (0.48%)	0 (0.00%)	0 (0.00%)	19 (0.45%)	*p* = 0.664
	Respiratory failure	16 (0.41%)	0 (0.00%)	1 (0.48%)	17 (0.41%)	*p* = 0.627
	Pleuritis	1 (0.03%)	0 (0.00%)	0 (0.00%)	1 (0.02%)	*p* = 1
Postoperative complications: any	None	3735 (94.60%)	23 (88.46%)	198 (94.74%)	3956 (94.57%)	*p* = 0.321
	Present	213 (5.40%)	3 (11.54%)	11 (5.26%)	227 (5.43%)	

p - Qualitative variables: chi-squared test or Fisher’s exact test. Quantitative variables: Kruskal–Wallis test + post-hoc analysis (Dunn’s test)

* Difference is statistically significant (*p* < 0.05)

** Multiple choice — percentages do not add up to 100

Cholelithiasis was the most frequent surgical indication in patients with a history of AP, but this was less frequent in patients with current active AP, with the most common indication for this group being cholecystitis (53.85%). However, cholecystitis was the least frequent surgical indication in the patients with a history of AP (20.10%). These differences were statistically significant. The percentage of laparoscopies was the highest in the group with no history of AP and was lowest in the group with active AP. The percentage of laparotomies was the highest in the group with active AP and lowest in the group with no history of AP. The percentage of laparoscopies with conversion was the highest in the group with a history of AP and lowest in the group with active AP. The surgical duration was significantly longer in the group with a history of AP than in the group with no history of AP. No significant differences in the postoperative complication rates, regardless of the type, were found among the groups.

Subgroup analyses according to AP severity and timing were performed; however, these findings should be regarded as descriptive given the small number of patients with active or severe AP. Among patients stratified by AP severity, 15.4% (four cases) of those with active AP had severe AP. Laparoscopic cholecystectomy was performed in two of these cases, and laparotomy in the other two. The percentage of laparoscopies was highest in the mild AP group (87.23%) and lowest in the severe AP group (77.78%), while laparotomy was most frequent in severe AP (22.22%) and least in mild AP. Conversion from laparoscopy to laparotomy occurred most often in mild AP and was not observed in moderate or severe AP (Table 2).

**Table 2:** Comparison between patients according to AP severity

Parameter		Mild AP (*N* = 47).	Moderate AP (*N* = 27)	Severe AP (*N* = 18)	Total (*N* = 92)	*p*-value
Indications for surgery **	Cholelithiasis	39 (82.98%)	21 (77.78%)	14 (77.78%)	74 (80.43%)	*p* = 0.829
	Inflammation	8 (17.02%)	7 (25.93%)	4 (22.22%)	19 (20.65%)	*p* = 0.66
	Polyp	0 (0.00%)	0 (0.00%)	0 (0.00%)	0 (0.00%)	*n/s*
	Tumor	0 (0.00%)	0 (0.00%)	0 (0.00%)	0 (0.00%)	*n/s*
	Other	0 (0.00%)	0 (0.00%)	0 (0.00%)	0 (0.00%)	*n/s*
Mode of operation	Elective	41 (87.23%)	21 (77.78%)	13 (72.22%)	75 (81.52%)	*p* = 0.306
	Emergency	6 (12.77%)	6 (22.22%)	5 (27.78%)	17 (18.48%)	
Type of operation	Laparoscopy	41 (87.23%)	23 (85.19%)	14 (77.78%)	78 (84.78%)	*p* = 0.016 *
	Laparotomy	1 (2.13%)	4 (14.81%)	4 (22.22%)	9 (9.78%)	
	Laparoscopy + conversion	5 (10.64%)	0 (0.00%)	0 (0.00%)	5 (5.43%)	
Duration of operation [min]	Mean (SD)	72.54 (33.62)	73.31 (33.56)	61.09 (24.41)	70.77 (32.07)	*p* = 0.423
	Median (quartiles)	55 (50–89)	65 (58.75–85)	50 (41–77.5)	60 (50–85)	
	Range	30–180	30–180	35–105	30–180	
	n	37	16	11	64	
Abdominal drainage	No	6 (12.77%)	1 (3.70%)	0 (0.00%)	7 (7.61%)	*p* = 0.214
	Yes	41 (87.23%)	26 (96.30%)	18 (100.00%)	85 (92.39%)	

*n/s - non significant*

**Fig. 1:**
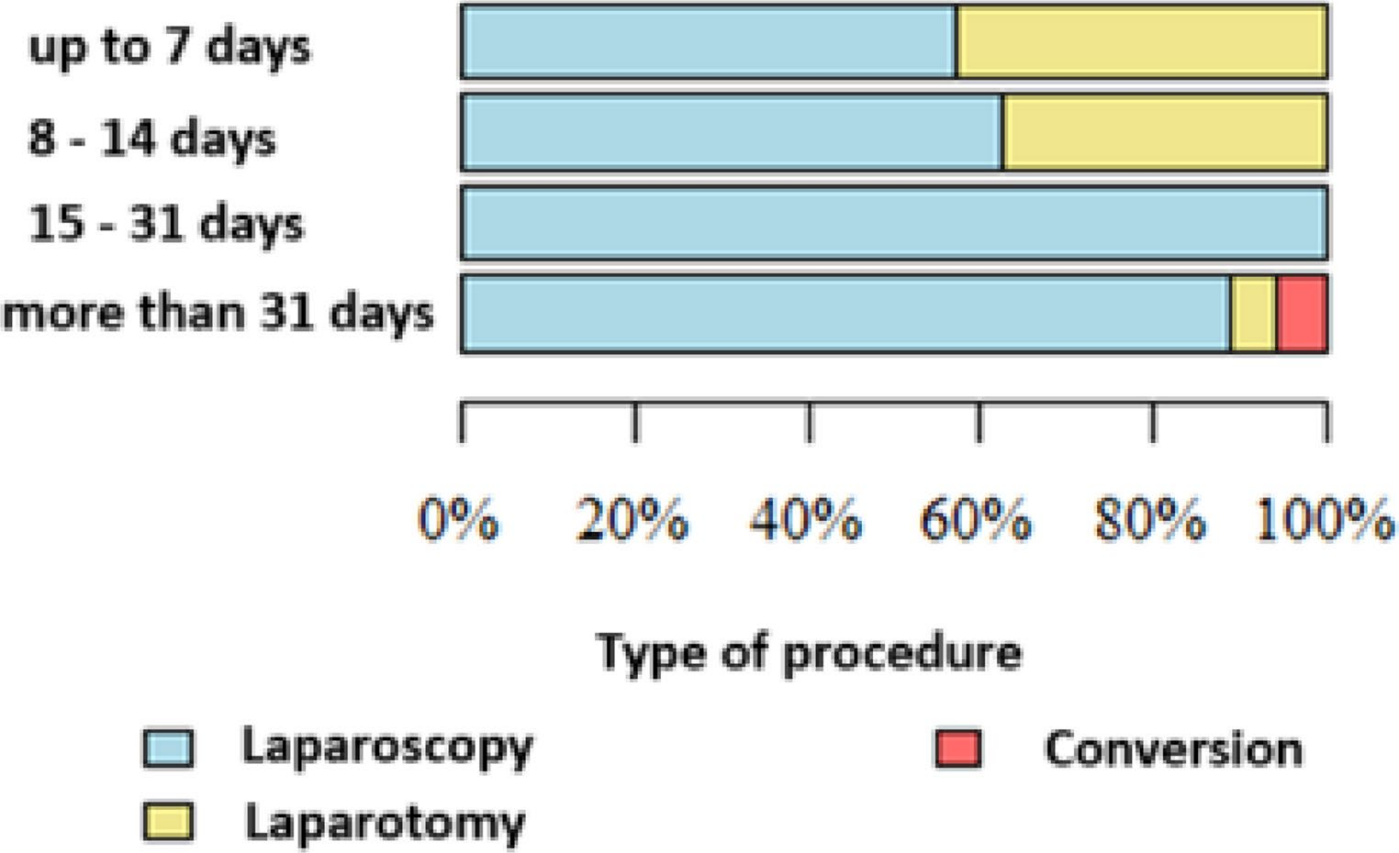
Type of surgery according to the time passed since the episode of acute pancreatitis

**Fig. 2:**
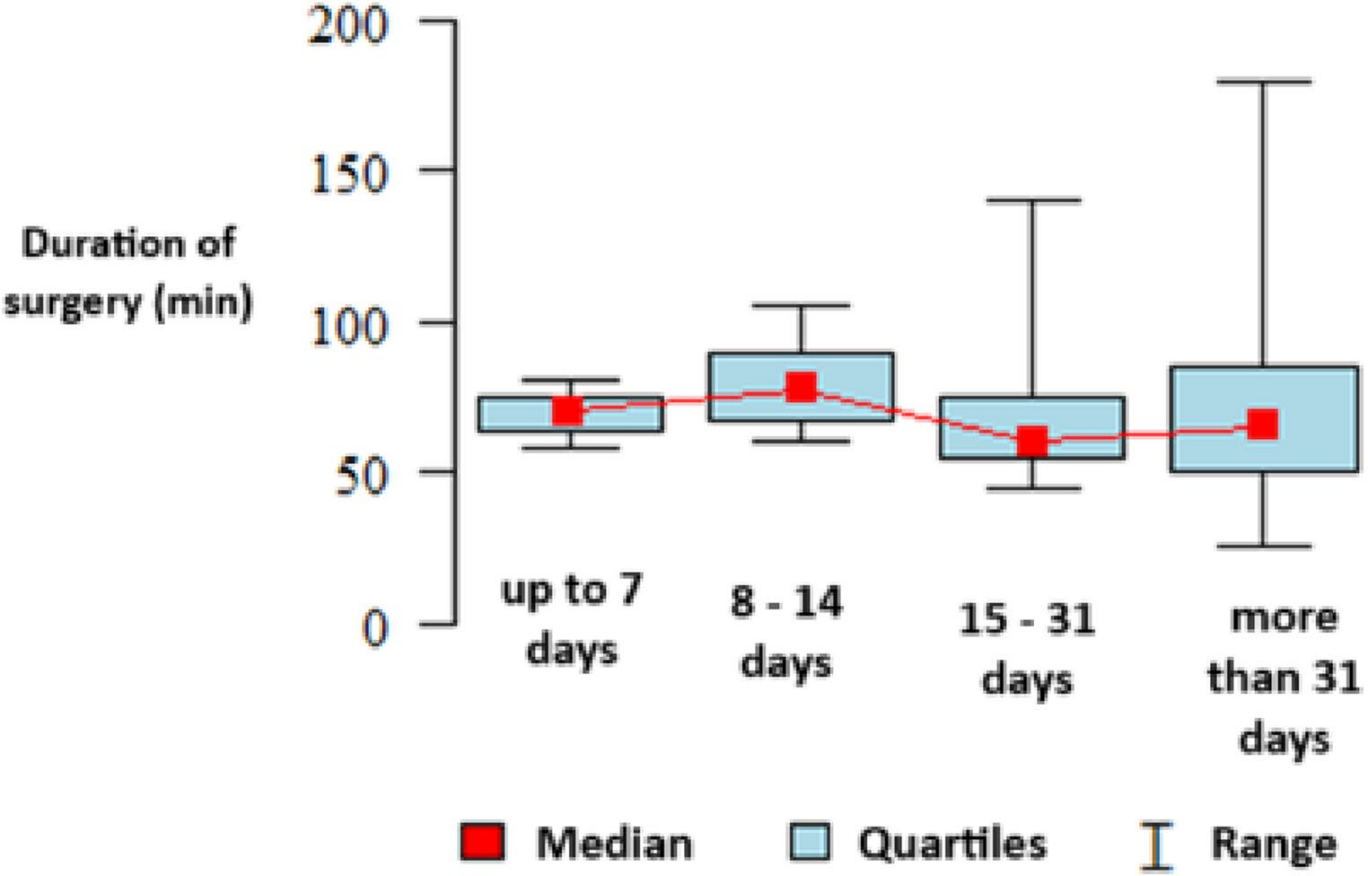
Duration of surgery according to the time elapsed since the episode of acute pancreatitis

The subgroup analysis of AP patients represents the core of our study, directly comparing outcomes of early versus delayed surgery in alignment with current clinical guideline debates. When stratified by time since the last AP episode, inflammation was the most frequent indication for surgery when performed within 7 days (71.43%), whereas cholelithiasis predominated when surgery was performed more than 31 days later (91.72%). The proportion of urgent surgeries was highest within 7 days and lowest beyond 31 days. Laparoscopy was most frequently performed when surgery took place 15–31 days after the AP episode, while laparotomy was most common within 7 days. Conversions from laparoscopy to laparotomy were seen most often when surgery was delayed beyond 31 days (Table 3; Figs. 1 and 2).

**Table 3:** Study data summarized according to the time between AP and surgery

Parameter		Time between AP and surgery					p-value
		Up to 7 days (*N* = 14)	8–14 days (*N* = 8)	15–31 days (*N* = 27)	More than 31 days (*N* = 145)	Total (*N* = 194)	
Indications for surgery **	Cholelithiasis	4 (28.57%)	4 (50.00%)	10 (37.04%)	133 (91.72%)	151 (77.84%)	*p* < 0.001 *
	Inflammation	10 (71.43%)	4 (50.00%)	19 (70.37%)	13 (8.97%)	46 (23.71%)	*p* < 0.001 *
	Polyp	0 (0.00%)	0 (0.00%)	0 (0.00%)	0 (0.00%)	0 (0.00%)	*n/s*
	Tumor	0 (0.00%)	0 (0.00%)	0 (0.00%)	0 (0.00%)	0 (0.00%)	*n/s*
	Other	0 (0.00%)	0 (0.00%)	0 (0.00%)	0 (0.00%)	0 (0.00%)	*n/s*
Mode of operation	Elective	2 (14.29%)	3 (37.50%)	25 (92.59%)	136 (93.79%)	166 (85.57%)	*p* < 0.001 *
	Emergency	12 (85.71%)	5 (62.50%)	2 (7.41%)	9 (6.21%)	28 (14.43%)	
Type of operation	Laparoscopy	8 (57.14%)	5 (62.50%)	27 (100.00%)	129 (88.97%)	169 (87.11%)	*p* < 0.001 *
	Laparotomy	6 (42.86%)	3 (37.50%)	0 (0.00%)	8 (5.52%)	17 (8.76%)	
	Laparoscopy + conversion	0 (0.00%)	0 (0.00%)	0 (0.00%)	8 (5.52%)	8 (4.12%)	
Duration of operation [min]	Mean (SD)	69.33 (11.02)	80 (19.58)	68.5 (23.64)	72.8 (31.13)	72.41 (29.74)	*p* = 0.742
	Median (quartiles)	70 (64–75)	77.5 (67.5–90)	60 (55–75)	65 (50–85)	65 (50–85)	
	Range	58–80	60–105	45–140	25–180	25–180	
	n	3	4	18	128	153	
Abdominal drainage	No	0 (0.00%)	1 (12.50%)	5 (18.52%)	8 (5.52%)	14 (7.22%)	*p* = 0.065
	Yes	14 (100.00%)	7 (87.50%)	22 (81.48%)	137 (94.48%)	180 (92.78%)	

*n/s - non significant*

Surgery in the period between 15 and 30 days after the AP episode was associated with the lowest risk of open surgery and shortest surgical duration, although the difference was not statistically significant (*p* = 0.742).

Regardless of the time when the surgery was performed and AP severity, the complications were not significantly different among the groups (Table 4).

**Table 4:** Postoperative complications among patients stratified according to the AP history status

Parameter		No AP (*N* = 3948) – A	Active AP (*N* = 26) – B	History of AP (*N* = 209) – C	Total (*N* = 4183)	*p*-value
Postoperative complications: surgical **	None	3791 (96.02%)	24 (92.31%)	198 (94.74%)	4013 (95.94%)	*p* = 0.264
	Infection of the surgical site	51 (1.29%)	0 (0.00%)	1 (0.48%)	52 (1.24%)	*p* = 0.653
	Bile leak	29 (0.73%)	1 (3.85%)	2 (0.96%)	32 (0.77%)	*p* = 0.143
	Postoperative hemorrhage	32 (0.81%)	1 (3.85%)	3 (1.44%)	36 (0.86%)	*p* = 0.093
	Other	33 (0.84%)	0 (0.00%)	3 (1.44%)	36 (0.86%)	*p* = 0.541
	Reoperation	13 (0.33%)	0 (0.00%)	2 (0.96%)	15 (0.36%)	*p* = 0.246
Postoperative complications: cardiological **	None	3897 (98.71%)	25 (96.15%)	207 (99.04%)	4129 (98.71%)	*p* = 0.425
	Heart rhythm disturbances	28 (0.71%)	0 (0.00%)	1 (0.48%)	29 (0.69%)	*p* = 1
	Cardiac failure	15 (0.38%)	0 (0.00%)	1 (0.48%)	16 (0.38%)	*p* = 0.604
	Infarction	8 (0.20%)	1 (3.85%)	0 (0.00%)	9 (0.22%)	*p* = 0.063
Postoperative complications: pulmonary **	None	3913 (99.11%)	26 (100.00%)	208 (99.52%)	4147 (99.14%)	*p* = 1
	Pneumonia	19 (0.48%)	0 (0.00%)	0 (0.00%)	19 (0.45%)	*p* = 0.664
	Respiratory failure	16 (0.41%)	0 (0.00%)	1 (0.48%)	17 (0.41%)	*p* = 0.627
	Pleuritis	1 (0.03%)	0 (0.00%)	0 (0.00%)	1 (0.02%)	*p* = 1
Postoperative complications: any	None	3735 (94.60%)	23 (88.46%)	198 (94.74%)	3956 (94.57%)	*p* = 0.321
	Present	213 (5.40%)	3 (11.54%)	11 (5.26%)	227 (5.43%)	

Only a small number of patients (e.g., 14 with a history of severe AP) were available for analysis, and therefore these findings should be interpreted as exploratory rather than definitive. All of them underwent surgical treatment at > 31 days after the completion of treatment for AP. Of them, two patients underwent laparotomy, whereas the remaining patients underwent laparoscopic surgery, with no significant differences between them and the other groups.

## Discussion

The inclusion of a non-AP comparator group was intentional, as it provides clinical context and allows quantification of how AP alters the operative course relative to standard cholecystectomy. Nevertheless, our subgroup analyses focused specifically on patients with AP to directly address the timing and severity questions central to clinical decision-making. The most clinically relevant analysis is the comparison within the AP cohort, where outcomes were stratified by surgical timing. This design directly addresses the ongoing question of early versus delayed cholecystectomy after AP raised in guideline discussions. Our findings indicate a trend toward surgery between 15 and 31 days being associated with higher rates of laparoscopic cholecystectomy and shorter operative duration. As these results were not statistically significant, they should be interpreted cautiously. We regard this as a signal rather than a definitive conclusion, consistent with prior reports, and emphasize the need for validation in prospective trials. In addition, inflammation was the most frequent indication for surgery during the course of active AP, whereas stones predominated when surgery was performed more than 31 days after an AP episode.

Nearly 6% of the world’s population suffers from cholelithiasis [16]. This prevalence rate is largely related to the lifestyle of the population and the ongoing epidemic of obesity, which, despite having reached a plateau of sorts in some developed countries, continues to increase globally in terms of the absolute number of patients with obesity [17]. Given that the occurrence of obesity, as well as rapid weight loss (owing to metabolic surgery or pharmacotherapy), is associated with the development of gallstones, the number of patients with complications of this condition, including the development of AP, is expected to increase.

A meta-analysis comparing early (< 72 h) and delayed (> 72 h) cholecystectomy in patients with mild AP was conducted by Yuan et al. [10]. They found that the risk of conversion to open surgery in early cholecystectomy was 3.81%, whereas that in delayed cholecystectomy was 3.26%. Our findings are consistent with the most recent IAP/APA revised guidelines [18] as well as the Japanese Guidelines [12] and TG18 [19], all of which recommend early cholecystectomy in mild biliary pancreatitis and delayed intervention in severe cases, emphasizing the need to tailor surgical timing to disease severity. Although our results suggest a trend favoring the 15–31 day window, they should be interpreted with caution given the retrospective design and limited sample size in severe AP [18].This is in accordance with our study results, where the highest percentage of procedures completed by using the minimally invasive method was observed between days 15 and 31 after the completion of AP treatment. No differences in postoperative complications rates were observed between early and delayed cholecystectomies in the previous study. However, some studies [20, 21] recommend that cholecystectomy be performed within 48–72 h after the onset of symptoms or even earlier in cases of mild AP [13]. However, most prognostic scales and prognostic factors are not sufficient to completely predict AP. Therefore, patients are potentially at risk for progression to severe AP with worsening prognosis. In our study, early cholecystectomy (performed up to 7 days) was associated with a higher percentage of open surgery. However, in a randomized trial by Davoodabadi et al. [21], no difference in surgical duration was observed between early and delayed surgeries post-AP episode. Similar results were also found in our study, where no significant difference was observed between the surgical durations in respect to the time elapsed since the last episode of AP. Our study as well as those mentioned above found no differences in the postoperative complication rates between the patient groups.

No clear evidence was also obtained with regard to the timing of cholecystectomy procedures in patients with a history of severe AP. Some researchers [22–24] recommend performing cholecystectomy at 6 weeks after completion of treatment for AP, except for cases with necrotic complications. In our study, 14 patients with a history of severe AP underwent cholecystectomy. All of them were operated at 31 days after the AP episode. No significant differences in postoperative complications were observed between this group of patients and those with less severe acute AP.

No significant differences in the rate of complications, either intraoperative or postoperative, were observed in our study among the different surgical times based on the period elapsed between the AP diagnosis and surgery. Some authors have focused on the risk factors of difficult cholecystectomy in patients after an AP episode [25]. They found that male sex is a risk factor for difficult cholecystectomy. However, in our study, difficult cholecystectomy was defined according to whether the patient was converted to open surgery or had prolonged operative time. Taking this into account, severe AP could be considered a risk factor for intraoperative complications, albeit with no effect on the postoperative complication rates.

Our study has some limitations. First, this is a retrospective study involving a relatively small number of patients with AP compared to the entire cholecystectomy cohort. Another limitation is that our cohort included patients without AP, which may appear to broaden the study beyond the scope of the early-versus-delayed debate. However, subgroup analyses were performed exclusively within the AP population, and these results were emphasized as the primary clinical message. Third, the inclusion of all surgical indications introduces heterogeneity, as conditions such as biliary colic, cholecystitis, or gallstone pancreatitis differ in urgency, management, and surgical risk. This approach was deliberate to ensure external validity and to reflect real-world surgical practice. To mitigate this limitation, we stratified the analysis by surgical indication, which allowed us to isolate patterns specifically attributable to acute pancreatitis. Finally, the retrospective classification of AP severity using the Atlanta criteria was limited by the available records, which did not always provide systematic information on the duration of organ failure or the presence of local complications. This weakens the precision of our severity stratification, and comparisons between mild, moderate, and severe AP subgroups should therefore be interpreted with caution. We recommend that large prospective multicenter trials be conducted to more precisely evaluate the impact of AP on cholecystectomy outcomes.

## Conclusions

Our data suggest that surgery performed between 15 and 31 days after completion of treatment for AP may be associated with more frequent laparoscopic approaches and shorter operative times. However, as these findings were not statistically significant, they should be interpreted cautiously. No differences were observed in postoperative complication rates across different timings, underscoring the need for prospective multicenter trials to define the optimal surgical window more precisely.

## Data Availability

No datasets were generated or analysed during the current study.
